# Evaluation of an Expert Guided Integrative Therapy Concept in Patients With Breast or Gynecological Cancer During Systemic Therapy

**DOI:** 10.1177/2515690X20949444

**Published:** 2020-08-18

**Authors:** Georg Schmidt, Sofia Mathes, Evelyn Klein, Marion Kiechle, Daniela Paepke

**Affiliations:** 1 Department of Gynecology and Obstetrics, Klinikum rechts der Isar and Comprehensive Cancer Center (CCCTUM), TU Munich, Germany

**Keywords:** complementary and alternative medicine (CAM), integrative medicine, integrative oncology, nutritional counseling, breast cancer, gynecological cancer, psycho-oncology

## Abstract

**Purpose.:**

Breast and gynecological cancer patients undergoing systemic therapy frequently request integrative therapy concepts. The potential of integrative therapy (IM) lies in minimizing side effects of conventional cancer treatments and therefore decreasing treatment delays. IM can help to improve patients’ physical and emotional well-being, optimizing health and quality of life as IM involves patients in their own treatment. A counseling service for integrative medicine concepts as an outpatient program was implemented in our cancer center in 2013.

**Methods.:**

In 2016 and 2017 144 breast and gynecological cancer patients were included into our specific IM program. The program comprises biological based complementary and alternative medicines (BB-CAM), a structured exercise therapy, manipulative and body-based practices, nutritional counseling, psycho-oncological and relaxing therapies. Therapists with additional specialization for IM, guide the treatment units. The program was evaluated via self-administered questionnaire.

**Results.:**

78% of the participating patients noticed an improvement by using BB-CAMs. 86% stated to feel better through participation in the structured exercise program. 74% profited from nutritional counseling and 91% from manual therapy. 93% of the patients treated with body compresses considered the application as soothing. The Bio-Frequency Sound Color Bed led to a relaxation in 96%. Psychological therapy improved coping with the disease in 70% of the patients.

**Conclusion.:**

Integrative oncology combines the best practices of conventional and complementary therapy, uniting them in a holistic concept. Data show that our integrative therapy concept is well accepted by the patients and that therapy- and disease-related side effects can be reduced.

## Introduction

As a part of the comprehensive cancer center, the department of gynecology and obstetrics, is specialized in the treatment of breast and gynecological cancer. The infrastructure provides the patient with an interdisciplinary therapy from the initial diagnosis to specific treatments such as surgery, chemotherapy, immunotherapy and radiotherapy in a single cancer center. Patients suffer from various symptoms from their cancer disease and conventional cancer therapies are often accompanied by serious side effects such as pain, fatigue, sleep disturbances, nausea and severe psychological disorders.

The well-informed patient is confronted with media offers such as guidebooks, self-help groups or online blogs informing about the diagnosis and the different potential treatments. Especially for complementary and alternative medicine (CAM) therapies during systemic treatment there is a flood of information presented by these media. Unfortunately, CAM methods are often used randomly and without informing the treating specialist. Thereby patients may generate negative effects through treatment interactions. Therefore, it is import to inform patients and actively advise proven CAM therapies or call attentions to detrimental effects.

It is the challenge of evidence-based medicine to spar with CAM to integrate scientifically validated complementary treatments into guidelines. Integrative oncology, which is generally understood, as a combination of complementary medicine therapies in conjunction with conventional cancer treatments. It has been defined in different ways, but there is no widely accepted definition. The term integrative oncology defines the combination between the best practices of conventional and complementary oncology.^1,2^ *The Society of Integrative Oncology (SIO)* uses the following definition for integrative oncology: “Integrative oncology is a patient-centered, evidence-informed field of cancer care that utilizes mind and body practices, natural products, and/or lifestyle modifications from different traditions alongside conventional cancer treatments. Integrative oncology aims to optimize health, quality of life, and clinical outcomes across the cancer care continuum and to empower people to prevent cancer and become active participants before, during, and beyond cancer treatment.^3^”

Looking at all cancer entities, studies showed that, depending on the country, more than 50% of cancer patients use complementary therapies during treatment.^4-9^ In breast cancer, especially young women with none metastatic disease seem to be interested in integrative oncology during cancer therapy.^10-13^ Different national and international cancer guidelines such as the German AGO (Arbeitsgemeinschaft Gynäkologische Onkologie) guideline for breast cancer try to integrate evidence-based CAM concepts to distance them from unconventional methods.

Until today, there is no requirement for certified gynecological or breast cancer center to include integrative oncology therapy recommendations into their therapy concepts. And furthermore, if integrative therapy concepts are implemented, it is unclear what elements they should contain. Due to these missing specifications, many different approaches of integrating IM into oncological treatment concepts exist. A review of integrative oncology programs by Seely et. al from 2012 tried to give an overview about CAM programs published worldwide. 29 programs of integrative oncology were included, most of them based in Untied States and the UK. Most of the offers were hospital-based and only 12 of 29 programs provided both conventional and complementary treatments in the same location. In most programs, mind-body medicine, massages, nutrition counseling and acupuncture were offered and only 3 programs offered exercise therapies.^14^

To meet the demand of IM offers for gynecological and breast cancer patients, the gynecological department founded a center for integrative gynecology and obstetrics (ZIGG, *Zentrum für Integrative Gynäkologie und Geburtshilfe; Center for integrative Gynecology and obstetrics*). Unlike as in other cancer centers, a consultation at our outpatient clinic regarding conventional systemic cancer treatment and integrative oncology concepts is offered by the same physician. During the initial assessment, the oncologist develops a therapy plan in consideration of the individual systemic therapy, the expected common side effects and also patients’ specific needs and requests. The therapies offered within the program, mostly took place in the same center. Some therapies, especially sports and manual therapy could be carried out near the patient´s homes. All therapies were conducted by trained therapist.

After having established the *ZIGG* program, we evaluated its acceptance and analyzed if patient’s needs where met. Also, an analysis if the therapy offers resulted in subjective improvement of each patient’s well-being was performed. The results of our survey are intended to improve the ZIGG program and provide a basis for further studies.

## Methods

During the years 2016 and 2017 144 patients with gynecological and breast cancer participated in our specific integrative medicine program (ZIGG) and were included in the study. A self-administered 47-item questionnaire was handed out to all patients at the end of the ZIGG program. It was designed to clarify the needs and interests of the women, as well as the acceptance of the offered therapies and applications. Furthermore, it queried if the program was able to improve the subjective quality of life as the current health condition and its change after participating in the ZIGG program was asked.

Within the ZIGG program, the patients were able to choose different IM elements based on individual medical recommendations and according to their needs and interests. Possible elements were:

Receiving a medical treatment plan (BB-CAM), external applications such as therapeutic body wraps (TBW), meditation on the bio-frequency sound bed, manual therapy, personalized fitness examination and instructions, nutritional consultation and consultation of a psycho-oncologist. The women attended at least 7 different therapy sessions within a 3-month period. The chosen IM elements could be carried out partly near their home areas.

At baseline of the program a consultation with a specialized gynecologist, who is certified in integrative and naturopathic medicine takes place. Depending on the systemic therapy and expected common side effects, an integrative medicine therapy plan including a biological based complementary medical treatment plan (BB-CAM) is developed and adjusted to the needs of the patient in regular follow-ups. Biologically based complementary and alternative medication (BB-CAM) includes herbs, phytotherapy, trace elements, and megavitamins/minerals. They are often already used via self-medication which entails the risk of interactions between the conventional cancer medications (CAM-drug interactions [CDIs]). Therefore, pre-existing BB-CAMs are documented and newly advised BB-CAMs are implemented in a plan which is checked for potential CAM-drug interactions.^15^

Additionally, external applications such as liver wrap, meditation on the bio-frequency sound bed, lymph therapy and kinesio-taping are offered by trained physio therapists and breast-care nurses. In addition to lymph therapy and conventional physiotherapy to treat the side effects of the cancer disease like lymphedema or pain, our therapists offer complementary treatments like massage and kinesio-taping. Due to the lack of qualified therapists the program did not offer acupuncture.

At the center for preventive and sports medicine the women receive an examination, to evaluate their level of fitness and to identify cardio-vascular risk factors. At a specialized rehabilitation center, the women are able to work on their physical performance under personalized fitness instructions by a trained physiotherapist.

In addition, the women receive a nutritional consultation with adjustment of eating habits. The Nutritional Counseling was performed according to the guidelines as set forth by the American Cancer Research Society and the Deutsche Krebsgesellschaft. Patients also have the opportunity to attend cooking classes learning special recipes and cooking methods to combat side effect such as nausea, vomiting and prevention of cachexia.

The consultation of a psycho-oncologist is another important part of the program and every woman is encouraged to take advantage of this offer. In addition, patient seminars for managing fatigue are provided.

### Bio-Frequency Sound Bed

The Bio-Frequency Sound Bed with color application was developed by the Buddhist monk Dokuho J. Meindl who is a physician for naturopathic medicine and psychotherapy. It is based on the theory of the 5 elements (earth, water, fire, wood and metal) of the traditional Chinese medicine. During the relaxation therapy, different music and colors are chosen individually for each patient. Every color is related to an organ or an emotion according to the TCM philosophy. The colors that are use are:

- red for fire associated with the heart and small intestine and the emotion overexcitation
- blue for water associated with the kidneys and bladder and the emotion anxiety and posttraumatic stress
- green for wood associated with the liver and gallbladder and the emotion anger and rage
- yellow for earth associated to the spleen and stomach and the emotion worry and brooding
- gray for metal associated to the lung and colon and the emotion grief and concern

The sound bed vibrates according to the chosen music and so the patient’s body is stimulated by deep frequencies. The idea is to focus on all senses of the patient to reach a deep state of relaxation.

### Wrap-Application

Therapeutic body wraps (TBW) belong to the so-called hydrotherapies, which are components of anthroposophical and naturopathic medicine. Due to lacking randomized data it belongs to empirical medicine.^16^ Liver wraps with achillea millefolium were the most commonly used TBW in our expert guided integrative therapy concept. It combines both heat and relaxation therapy and aims to reduce fatigue and ease anxieties. A liver wrap with Achillea millefolium (common yarrow) has anti-inflammatory and antispasmodic attributes. The liver function can be beneficially influenced, e.g. sleep disorders, depressed mood, lethargy and also liver capsule pain.^17^ Trained nurses carry out the wrap-application and additionally instruct the patients to perform the procedure themselves at home. The wrap consists of 3 cotton wool sheets. The inner sheet is soaked in hot achillea millefolium tea and is then directly applied to the liver region. Then the patients are wrapped in 2 further dry sheets and covered with a blanket.

The participants of the evaluation provided informed consent. The study was planned with the advice of the Ethics Committee.

## Results

In 2016 and 2017 144 patients who consulted the outpatient service for integrative therapy were admitted to the ZIGG program. Every patient who has been treated in our department for breast or gynecological cancer was offered to join the ZIGG program. In 2016 43% of our patients with breast cancer under systemic therapy joined the program. After completing the ZIGG program, the women were asked to fill out the questionnaire. The response rate was 82.6% (n = 119). The median age of responding women was 55 years.

All but 1 patient received systemic oncological treatment. 40% of the patients (n = 47) were in an adjuvant, 35% (n = 41) in a neoadjuvant and 25% (n = 30) in a palliative therapy situation.

84% of the responding women had breast cancer, 13% had ovarian cancer and 3% presented with other gynecological cancers. Table 1 shows the spread of integrative therapeutic approaches among the tumor entities.

**Table 1.** Comparison of the Performed IM Therapies and Tumor Entities.

Use of integrative therapeutic approaches and check up	All respondents (n = 119)	Women with breast cancer (n = 100)	Women with ovarian cancer (n = 15)	Women with other gynecological cancer (n = 4)
Sports medical check-up (%)				
	55	56	53	25
	(n = 65)	(n = 56)	(n = 8)	(n = 1)
Use of sports therapy (%)				
at TUM (Technische Universität München)	35	37	27	25
	(n = 42)	(n = 37)	(n = 4)	(n = 1)
external	19	17	33	25
	(n = 23)	(n = 17)	(n = 5)	(n = 1)
Use of nutritional counseling (%)				
at TUM	56	59	53	0
	(n = 67)	(n = 59)	(n = 8)	(n = 0)
external	1	1	0	0
	(n = 1)	(n = 1)	(n = 0)	(n = 0)
Use of body compresses (%)				
at TUM	77	77	73	100
	(n = 92)	(n = 77)	(n = 11)	(n = 4)
external	3	2	13	0
	(n = 4)	(n = 2)	(n = 2)	(n = 0)
Use of manual therapy (%)				
at TUM	30	33	20	0
	(n = 36)	(n = 33)	(n = 3)	(n = 0)
external	8	10	0	0
	(n = 10)	(n = 10)	(n = 0)	(n = 0)
Use of Bio-frequency sound bed (%)				
at TUM	74	72	80	100
	(n = 88)	(n = 72)	(n = 12)	(n = 4)
Use of psychological therapy (%)				
at TUM	61	61	73	25
	(n = 73)	(n = 61)	(n = 11)	(n = 1)

The utilization of the individual therapeutic offers was also evaluated by 3 different age groups (Age ≤ 50, Age 51 - 60, Age 61 ≤) displayed in Table 2.

**Table 2.** Comparison of the Performed IM Therapies in the Different Age Groups.

Use of integrative therapeutic approaches	All respondents (n = 119)	Age ≤50 (n = 37)	Age 51-60 (n = 51)	Age ≥61 (n = 31)
Sports medical check-up (%)				
	55	73	43	52
	(n = 65)	(n = 27)	(n = 22)	(n = 16)
Use of sports therapy (%)				
at TUM (Technische Universität München)	35	43	29	36
	(n = 42)	(n = 16)	(n = 15)	(n = 11)
external	19	22	16	23
	(n = 23)	(n = 8)	(n = 8)	(n = 7)
Use of nutritional counseling (%)				
at TUM	56	76	43	55
	(n = 67)	(n = 28)	(n = 22)	(n = 17)
external	1	3	0	0
	(n = 1)	(n = 1)	(n = 0)	(n = 0)
Use of body compresses (%)				
at TUM	77	92	71	71
	(n = 92)	(n = 34)	(n = 36)	(n = 22)
external	3	0	2	10
	(n = 4)	(n = 0)	(n = 1)	(n = 3)
Use of manual therapy (%)				
at TUM	30	35	35	16
	(n = 36)	(n = 13)	(n = 18)	(n = 5)
external	8	8	14	0
	(n = 10)	(n = 3)	(n = 7)	(n = 0)
Use of Bio-frequency sound bed (%)				
at TUM	74	87	65	74
	(n = 88)	(n = 32)	(n = 33)	(n = 23)
Use of psychological therapy (%)				
at TUM	61	76	59	48
	(n = 73)	(n = 28)	(n = 30)	(n = 15)

Table 3 shows the subjective effect on the patients’ well-being of each element of the program as BB-CAMs, sports therapy, manual therapy and psychological therapy. Wraps were the most commonly used treatment with a high degree of acceptance in all age groups (92% in age group ≤ 50, 71% in age group 51- 60, 71% in age group ≥ 61).

Our data confirm a higher participation of younger women in integrative oncology programs (e.g. for sports therapy 43% vs 29% vs 36%). In contrast patients between 51 and 60 years (second age group) used all ZIGG offers the least and were less likely to use other therapeutic offers outside the ZIGG program.

**Table 3.** Effect on Health Condition.

Integrative therapeutic approaches	Health condition has been improved	No change in health condition	Health condition has worsened
CAM (%)	80	16	4
(n = 116)	(n = 93)	(n = 19)	(n = 4)
Sports therapy (%)			
All	88	12	0
(n = 59)	(n = 52)	(n = 7)	(n = 0)
at TUM (Technische Universität München)	86	14	0
(n = 42)	(n = 36)	(n = 6)	(n = 0)
external	94	6	0
(n = 17)	(n = 16)	(n = 1)	(n = 0)
Manual therapy (%)			
All	93	7	0
(n = 44)	(n = 41)	(n = 3)	(n = 0)
at TUM	91	9	0
(n = 35)	(n = 32)	(n = 3)	(n = 0)
external	100.0	0	0
(n = 8)	(n = 8)	(n = 0)	(n = 0)
Psychological therapy (%)	86	14	0
(n = 72)	(n = 62)	(n = 10)	(n = 0)

### BB-CAM Medication

**Figure 1.**
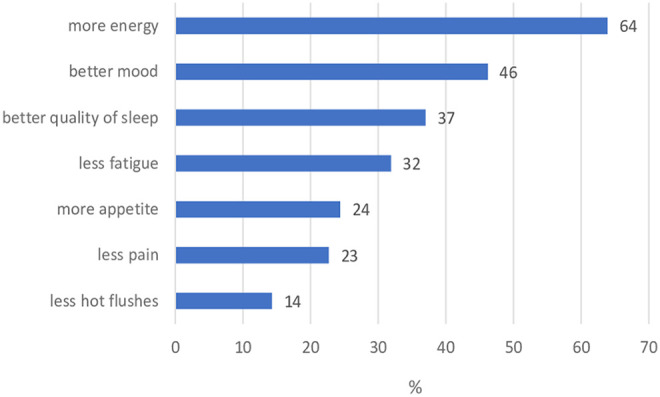
Type of improvement through BB-CAM medication.

Every woman (n = 119) surveyed received a BB-CAM medication plan during their systemic therapy. Depending on the type of cancer, the patient´s individual health status, their actual systemic treatment, pre-existing and expected side effects of their treatments and preferences of the patient, specific BB-CAMs were prescribed by a specialized gynecologist. Prescribed medications were mistletoe, selenium acid, vitamin D3 and further individual homeopathic medication. 78% of the patients (n = 93) reported a subjective improvement in their well-being while taking any BB-CAMs. It was examined what kind of subjective change of the patient’s side effects could be achieved. Most of the patients (77%) attributed the greatest benefit to the mistletoe therapy. See the subjective evaluation of the patients in Figure 1.

### Wrap Application

92 of 119 patients (77%) received therapeutic body wraps. During the survey period, 623 wraps were applied. Minimum application was 1, maximum were 27 wraps per patient. Liver wraps with achillea millefolium were the most common type of TBW (95%), more rarely abdominal wrap with oxalis (4%) and pulse wrap with lavandula angustifolia (2%). Through the application of the wraps 76% of the patients described the status of relaxation as significant, 22% as moderate and 2% as light. The patients described a loosening and warming effect and thereby felt fully relaxed. General 93% rated the TBW as a subjective success. 53% continued the applications as recommended at home.

### Bio-Frequency Sound Bed

74% of the patients (n = 88) had a bio-frequency sound bed therapy. The most common color chosen by the physician was blue (74%), followed by green (10%), yellow (8%), red (6%) and gray (1%).

96% of the patients could reach a deep relaxed state and meditative tranquility. 40% stated that the deep relaxed state and meditative tranquility lasted for the time of the bio-frequency sound bed therapy (30 min). 52% stated that the calm mood lasted for the rest of the day. Only 3% could not reach a relaxed state.

### Psycho-Oncological Therapy

**Figure 2.**
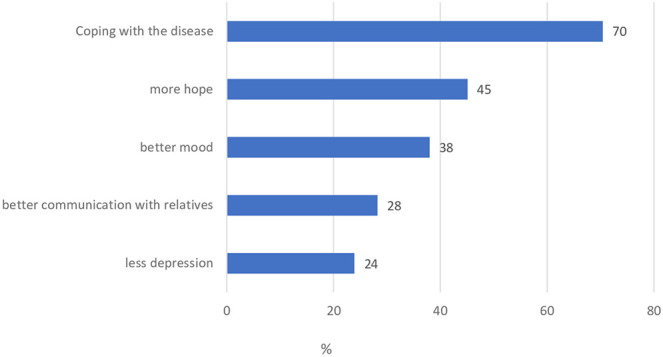
Effects through psycho-oncological therapy.

During the survey period, the psychologist of the gynecological cancer center documented 173 psycho-oncological therapy sessions with 73 patients (61%). 86% of the patients achieved a subjective improvement of their current condition. Figure 2 shows the effects of the psychological therapy sessions.

### Nutritional Counseling

**Figure 3.**
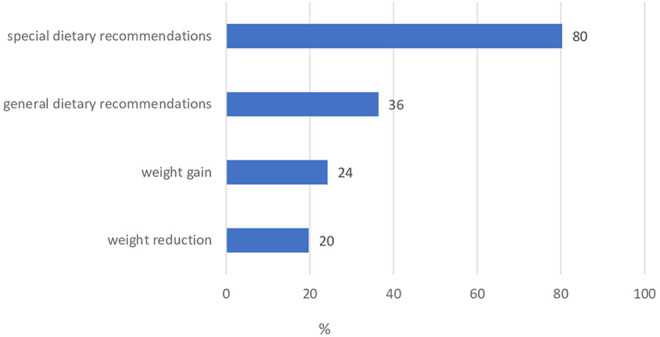
Patient's expectations of nutritional counseling.

56% (n = 67) of the patients took part in nutritional counseling. 54 nutritional counseling sessions were carried out at the Center for Prevention and Sports Medicine. The following chart shows the patient´s expectations of nutritional counseling (Figure 3).

In 71%, the patient´s personal expectations were met. 79% found the nutritional advice helpful.

### Exercise Program

At the Center for Prevention and Sports Medicine, 126 appointments were attended by the patients (minimum per patient: 1, maximum per patient: 7, median: 2). These included an electrocardiogram, cardiac echocardiography, anthropometry and bicycle ergometry with lactate diagnostics. 65 women (55%) had a baseline examination at the Center for Prevention and Sports Medicine to evaluate their health status and performance level. After the examination 42 of the patients (35%) participated in the centers exercise program and 23 of the patients (19%) attended an external sports program.

The measurement of anthropometric parameters such as body mass index (BMI) and body fat were offered as part of the sports medicine baseline examination. 50 women accepted the measurement. The BMI and body fat increased with age. In addition, breast cancer patients had a higher BMI and a significantly higher body fat percentage than ovarian cancer patients (Table 4).

**Table 4.** Body-Mass-Index and Body Fat Percentage in Different Age Groups and Different Types of Cancer.

	Body mass index, $\bar{\text{x}}$ ± SD	p-value	Body fat percentage, $\bar{\text{x}}$ ± SD	p-value
All				
(n = 50)	23.62 ± 4.29		26.24 ± 5.14	
Age group (%)				
Age ≤ 50		0.197		0.176
(n = 21)	23.02 ± 3.32		24.72 ± 5.01	
Age 51 - 60				
(n = 16)	22.90 ± 4.06		26.63 ± 4.87	
Age 61 ≤				
(n = 13)	25.47 ± 5.59		28.32 ± 5.35	
Type of cancer (%)				
Breast cancer		0.451		0.007
(n = 43)	23.87 ± 4.14		26.85 ± 4.90	
Ovarian cancer				
(n = 6)	22.43 ± 5.63		19.75 ± 3.74	

**Figure 4.**
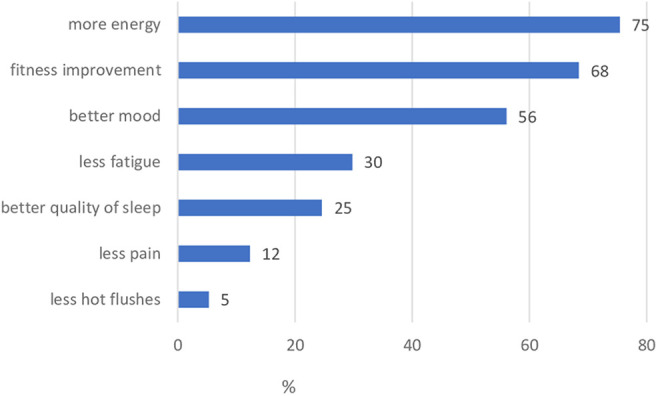
Type of improvement through the structured rehabilitation exercise program.

By participating in the structured rehabilitation exercise program, 86% of the patients achieved a subjective improvement of their health state. Reasons for not participating in the structured exercise program in our cancer center were, among other things, a too long distance to the sports center and already existing gym membership (Figure 4).

### Manual Therapy

195 lymphatic drainages were performed during the study. 30% (n = 36) had physiotherapy at the cancer center, 8% (n = 10) attended external physiotherapist. With 89%, lymphatic drainage was the most common form of manual therapy, followed by scar therapy (23%), massage (6%) and kinesio-taping (6%). Overall, manual therapy improved the subjective condition of 91% of in-house treated patients.

### Rating of the ZIGG Program

The patients were asked to give an overall evaluation of the ZIGG program using the German school grade scale from 1 to 6 (1 = best to 6 = worst). The ZIGG program was rated with an overall grade of 1.47, which shows a high satisfaction. There are no significant differences between the different age groups and cancers types.

## Discussion

In our survey, we evaluated an expert guided integrative therapy concept at an University Hospital for women with breast or gynecological cancer undergoing systemic cancer therapy. It intends to show, if our integrative medicine concept can meet our patients interests and growing demands for integrative health approaches. The survey is based on a 47-item questionnaire in which patients subjectively assessed the program.

The high interest in and need for information about integrative therapy concepts among women with breast and gynecological cancer is well examined in literature.^11,18-20^ In Germany, an AGO study managed to show that the growing interest of physicians and patients in integrative medicine does not match the actual offer of integrative treatment approaches in evidence-based cancer centers.^21^ Due to the sparse offers of integrative therapy concepts in cancer centers, patients often approach not qualified CAM providers.^22-25^ Furthermore, in only 1% recommendations for integrative medicine therapies were advised by physicians.^22,26^ This is contrary to patient´s demands, who desire a consultation from their oncologist and wish an extensive and high qualitative counseling interview.^11,27^

At the gynecological and breast cancer center every patient with breast or gynecological cancer under adjuvant, neoadjuvant or palliative therapy is able to participate in the ZIGG program.

Our data showed that this specialized program was preferably taken advantage of by patients in an early and none metastasized state of disease (75% adjuvant and neoadjuvant setting). This is congruent with published data.^11,26,28^ While other data showed, that especially young women with metastatic breast cancer use CAM,^7^ only 25% of our surveyed patients where in a palliative treatment situation. Interestingly, the M D Anderson Cancer Center used complementary treatment approaches especially during progression or failure of primary treatment.^29^

116 women received a BB-CAM plan during systemic treatment. 80% of the patients (n = 93) reported a subjective improvement in their state of health. Most women felt more energy and were in a better mood. Regarding the high number of women, who asked for complementary medication, we want to emphasize that the treatment plan should be development by physicians, which are trained in systemic cancer treatment as well as common complementary therapy concepts. Interaction between CAM medication and systemic cancer treatment can reduce efficacy and increase toxicity. Phytotherapeutics such as St. John's wort could decrease the plasma level of chemotherapeutics, which may have a negative impact on treatment outcome.^30^ Other phytotherapeutics such as supportive mistletoe are well examined, well tolerated and are able to improve the quality of life during cancer treatment.^31-34^ In context of new treatments such as immunomodulation, phytotherapeutics like Viscum album have to be tested in vitro for interactions.^35^ Because of the known potential for interactions, every individual CAM treatment plan is checked for interactions with the common systemic cancer therapy at our cancer center.^28^

Patients assigned to exercise and nutritional counseling within the ZIGG program were sent to the center of preventive and sports medicine. The physicians develop individualized training plans for moderate sport and nutrition recommendations. There is a lot of evidence in favor of targeted exercise prescription after an examination of patients’ cardiovascular health and fitness level during cancer therapy.^36-39^ 85% of the women who participated in the structured exercise program reported an improvement of their condition. An individualized training plan can avoid cardiovascular side effects and quitting due overstraining or frustration.

Furthermore, we could show, that 74% of the women profited from nutritional counseling. The consultation serves to identify malnutrition or signs for sarcopenia and to give advice regarding dietary supplements and nutrition during chemotherapy. In advanced ovarian cancer, baseline sarcopenia is a prognostic factor. Therefore, the identification of sarcopenic patients and nutritional education might improve outcome.^40^ Survivors of breast cancer have a higher risk of cardiovascular and metabolic disease which again increases the risk of recurrent disease.^41,42^ *De Cicco et al.* reviewed existing literature and showed the evidence for nutritional counseling and supplementation with dietary constituents for breast cancer patients. 112 pertinent articles point out that nutritional intervention might improve overall survival and be helpful in reducing drug related side effects.^43^ Patients are confronting their physicians with requests for intermittent fasting, but the current state of science does not allow a general recommendation for short time fasting shortly before and after chemotherapy. Small pilot trials with 10 and 13 women showed a reduction of side effects like fatigue and reduced hematological toxicity. Still larger, randomized trials have yet to examine the effect and safety of fasting during chemotherapy.^44,45^

The German breast and gynecological cancer guidelines recommend psycho-oncological support for affected patients. It is part of the “National Cancer Plan” in Germany and a requirement to be certified as a breast and gynecological cancer center. Particularly pre-menopausal breast cancer survivors are at an increased risk of psychological morbidity.^46^ In our study, 61% of the patients had psycho-oncological counseling within the ZIGG program at our cancer center. This is a high participation rate compared to participation rate (42%) outside the ZIGG program. Additionally, every woman treated with a cancer diagnose is screened for the demand for psychological support (FBK-R10 questionnaire). The FBK-R10 questionnaire is a psychometrically evaluated test that measures psychosocial distress. It consists of 10 typical cancer related stress situations, each of which is answered according to relevance (applies / does not apply) and stress intensity. While 20-70% women who survived breast cancer need psychological support and the prevalence of anxiety and depression in this patient group is very high, a survey showed that 13% of participating German breast cancer centers are not offering psycho-oncological support by permanent employees.^47-49^

Comparing the ZIGG program with other integrative oncology programs in Germany breast and gynecological cancer centers you will find some differences. At Klinikum Essen-Mitte, the integrative oncology program is offered within a cooperation of the Breast Center and the Department of Internal and Integrative Medicine.^2^ However, the ZIGG program pursues a single center based, holistic approach. An advantage of this might be the formation of a stronger relation between patient and physician, who guides the women through diagnostics, chemotherapy, integrative therapy and surgery. On the other hand, the advantage of the cooperating program at Klinikum Essen-Mitte could be the ongoing exchange of expertise of the physicians and therapists at the Department of Internal and Integrative Medicine, who benefit from their experience in integrative therapy from other disease pattern.

Another example for a well implemented integrative oncological concept in a German breast cancer center is the Hospital Havelhöhe. Unlike the University Hospital of Technical University Munich, Havelhöhe is an anthroposophical Hospital. The aim of anthroposophical medicine is to improve quality of life and salutogenesis, with treatments like movement therapies, mind-body and art therapy and non-pharmacological interventions.^50-54^ Through the anthroposophical orientation of the entire certified hospital the professionals of the breast cancer center have a high experience in integrative oncology confirming the specified requirements of an evidence based, certified breast cancer center.

Concerning the financial cost and reimbursement, patients paid the BB-CAMs and utensils for the wraps out of their pocket. The outpatient clinic visits and the therapeutic applications were written off by the university and in parts through insurance reimbursement. The financing of the IO treatment differs in the different health systems. In the U.S. 21% of the IO costs were payed out of the pocket. The total costs for 1 year ranged between 1594$ for early stage breast cancer and more than 5000$ for stage 3 and 4 breast cancer patients.^55^ Further research and evidence in the field of integrative oncology may increase the reimbursement by insurance and improve the access to IO for more patients.

## Conclusion

The results of our study show that the integration of an expert guided integrative therapy concept in patients with breast or gynecological cancer during systemic therapy is well accepted by the patients and can improve patient´s subjective health condition. However, there are further randomized, prospective trials needed to objectify the positive impact of integrative oncology concepts in women with breast and gynecological cancer and to develop evidence-based treatment guidelines.

## Supplemental Material

Supplemental Material, Paper_ZIGG_JEBIM_questionnaire - Evaluation of an Expert Guided Integrative Therapy Concept in Patients With Breast or Gynecological Cancer During Systemic Therapy Click here for additional data file. Supplemental Material, Paper_ZIGG_JEBIM_questionnaire for Evaluation of an Expert Guided Integrative Therapy Concept in Patients With Breast or Gynecological Cancer During Systemic Therapy by Georg Schmidt, Sofia Mathes, Evelyn Klein, Marion Kiechle and Daniela Paepke in Journal of Evidence-Based Integrative Medicine
