# Supplementary material for: Evaluation of an Expert Guided Integrative Therapy Concept in Patients With Breast or Gynecological Cancer During Systemic Therapy
Source: J Evid Based Integr Med. 2020 Aug 18;25:2515690X20949444. doi: 10.1177/2515690X20949444 (PMC7436788; doi:10.1177/2515690X20949444)
Supplement: Supplemental Material, Paper_ZIGG_JEBIM_questionnaire - Evaluation of an Expert Guided Integrative Therapy Concept in Patients With Breast or Gynecological Cancer During Systemic Therapy [file Paper_ZIGG_JEBIM_questionnaire.pdf]

Evaluation of the ZIGG Program

I General Questions

1. How would you describe your current state of health?

❒ excellent ❒ very well ❒ well ❒ not well ❒ poor

II Questions about the complementary medications.

2. Do the complementary medications affect your health status?

My health status ❒ has been improved (please continue with question 2a)

❒ did not change

❒ became worse (please continue with question 2b)

2a ❒ more energy ❒ more appetite

❒ less hot flashes ❒ better sleep ❒ better mood ❒ less pain ❒ less fatigue

❒ others: ____

2b ❒ less energy ❒ fever

❒ worse sleep ❒ more pain ❒ worse mood

❒ others:

____

3. Has there been any reduction of side effects from chemotherapy, anti-hormonal therapy or radiotherapy since you are under complementary medication?

❐ yes ❐ no ❐ I don’t know

4. To which kind of complementary medication do you attribute the biggest benefit?

❐ mistletoe ❐ selenium ❐ vitamins ❐ others: ____

III Questions about sports therapy:

5. Did you have an initial examination at the centre for preventive and sports medicine (EKG, cardiac examination etc.)?

❐ yes ❐ no

6. Did you participate in the structured exercise program?

❐ yes ❐ no, reason: ________________________________

6a. How often did you participate in the exercise program?

❐ once a month ❐ once a week ❐ 2-3x/month

6b. How long did you participate in the exercise program?

❐ 1 month ❐ 2-3 months ❐ > 3 months

6c. Has there been any effect of the exercise program on your condition?

My condition ❒ has improved (please continue with question 7d)

❒ has been the same

❒ has gotten worse (please continue with question 7e)

6d ❒ more energy ❒ improvement of fitness

❒ less hot flashes ❒ better sleep ❒ better mood ❒ less pain ❒ less fatigue

❒ others: ____

6e Reasons for worsening: ____

IV Questions about nutritional counselling

7. Did you receive a nutritional counselling at the centre for preventive and sports medicine?

❒ yes ❒ no

8. How often did you receive the consultations?

❒ once ❒ twice ❒ >twice

9. What was your personal goal for nutritional counselling?

❒ weight reduction ❒ weight gain ❒ general recommendations for nutrition ❒ special recommendation for nutrition during oncological therapy

10. Did you achieve your personal goal?

❒ yes ❒ no

11. Do you think the nutritional counselling was helpful? ❐ yes ❐ no, reasons: ____

V Questions about the special cooking lessons

12. Did you participate in the special cooking lessons?

❐ yes ❐ no

13. What was your personal goal for the cooking lessons?

❐ weight reduction ❐ weight gain ❐ general recommendations for nutrition ❐ special recommendation for nutrition during oncological therapy

14. Did you achieve your personal goal?

❐ yes ❐ no

15. Do you think the cooking lesson was helpful? ❐ yes ❐ no, reasons: ____

VI Questions about wrap applications

16. Did you receive wrap applications?

❐ yes (please continue with question 17) ❐ no

(please continue with question 33)

17. What kind of wrap application did you receive?

❒ liver wrapping ❒ abdominal wrapping ❒ pulse wrapping

❒ ointment application ❒ others: ______________________________

18. Did the wrapping improve your well-being?

❒ yes

19. ❒ just during application ❒ for one day ❒ for 1 week ❒ > 1 week

❒ I don’t know

❒ no

20. How would you describe the degree of relaxation during the wrap-applications? ❒ high ❒ moderate ❒ low

21. How would you describe the proportion of wrap application on the effect of the whole ZIGG Program? ❒ high ❒ moderate ❒ no effect ❒ I don’t know

22. Would you evaluate the wrap application as a success? ❒ yes ❒ no ❒ I don’t know

23. Did you continue wrap applications at home?

❒ yes ❒ as recommended from the therapist ❒ less often than recommended ❒ no, reasons: ____

24. How would you rate the competence of the nurses who applied the wrap application?

❒ very good ❒ good ❒ satisfactory ❒ sufficient ❒ inadequate

VII Questions about the Bio-Frequency Sound Bed

25. Which combination of colours did you choose?

❒ red ❒ blue ❒ green ❒ yellow ❒ grey

26. How would you rate the degree of relaxation and tranquillity while you lay on the sound bed?

❒ high ❒ moderate ❒ low ❒ no relaxation

27. How would you rate the effect of the lights?

❒ very good ❒ good ❒ satisfactory ❒ sufficient ❒ inadequate

28. How would you rate the effect of the sounds?

❒ very good ❒ good ❒ satisfactory ❒ sufficient ❒ inadequate

29. How would you rate the effect of the vibration?

❒ very good ❒ good ❒ satisfactory ❒ sufficient ❒ inadequate

30. How would you rate the therapy room?

❒ very good ❒ good ❒ satisfactory ❒ sufficient ❒ inadequate

31. Could you reach a deep relaxed state and meditative tranquillity.?

❒ yes ❒ just during application ❒ for one day ❒ for 1 week ❒ > 1 week

VIII Questions about psycho-oncological therapy

32. Before you joined the ZIGG Program, did you get any psycho-oncological support?

❒ yes ❒ within Klinikum Rechts der Isar ❒ external provider ❒ no

33. How often did you received psycho-oncological therapy?

❒ one a month ❒ once a week ❒ 2-3x/week

34. How long did you participate in the psycho-oncological therapy?

❒ one month ❒ 2-3 month ❒ > 3 month

35. Did the psycho-oncological therapy affect your current condition? My condition ❒ has improved ❒ is the same ❒ has gotten worse

35a ❒ less depression ❒ more hope

❒ better coping with the disease ❒ better mood ❒ better communication with family and friends ❒ others: ____

35b Reasons for worsening: ____

IX Questions about manual therapy (lymphatic drainage, scar therapy, massage, kinesio-taping)

36. Did you receive any manual therapy within the Klinikum Rechts der Isar?

❒ yes ❒ no

37. What kind of therapy did you receive?

❒ lymphatic drainage ❒ scar therapy ❒ kinesio-taping ❒ massage

38. How often did you received manual therapy?

❒ one a month ❒ once a week ❒ 2-3x/week

39. How long did you participate in the manual therapy?

❒ one month ❒ 2-3 month ❒ > 3 month

40. Did the psycho-oncological therapy affect your current condition? My condition ❒ has improved ❒ is the same ❒ has gotten worse

X. Questions about fatigue training

41. Did you participate in the fatigue training at the Klinikum Rechts der Isar?

❒ yes ❒ no

42. Do you think the training was helpful? ❒ yes ❒ no

43. Do you think the recommendations from the fatigue training were helpful for you?

(1 = best, 6 = worst)

1 - - - - - - - 2 - - - - - - - 3 - - - - - - - 4 - - - - - - - 5 - - - - - - - 6

44. How would you rate the quality of the training?

(1 = best 6 = worst)

1 - - - - - - - 2 - - - - - - - 3 - - - - - - - 4 - - - - - - - 5 - - - - - - - 6

45. How would you rate the offer of the ZIGG Program

(1 = best, 6 = worst)

1 - - - - - - - 2 - - - - - - - 3 - - - - - - - 4 - - - - - - - 5 - - - - - - - 6

46. How strong could the ZIGG Program satisfy your need?

(1 = best, 6 = worst)

1 - - - - - - - 2 - - - - - - - 3 - - - - - - - 4 - - - - - - - 5 - - - - - - - 6

47. Further comments, criticism, wishes

____

______

____

______

____

______

____

______
